# A virus becomes a global concern: research activities on West-Nile virus

**DOI:** 10.1080/22221751.2023.2256424

**Published:** 2023-09-13

**Authors:** Doris Klingelhöfer, Markus Braun, Isabelle M. Kramer, Friederike Reuss, Ruth Müller, David A. Groneberg, Dörthe Brüggmann

**Affiliations:** a Institute of Occupational, Social and Environmental Medicine, Goethe University Frankfurt, Frankfurt, Germany; b Unit Entomology, Institute of Tropical Medicine, Antwerp, Belgium

**Keywords:** Culex, mosquitos, vector-borne diseases, emerging pathogens, infectious diseases

## Abstract

Currently, West-Nile virus (WNV) is spreading worldwide to colder regions due to climate change. Human mortality and morbidity are prevalent and steadily increasing, associated with costs to public health systems. Therefore, the question of the impact of scientific engagement arises. What trends, barriers, and incentives for research related to global burdens are important in this context? To answer these questions, this study provides detailed insights into the publication patterns of WNV research and interprets them using several parameters, such as absolute and relative publication indices and socioeconomic and epidemiological characteristics. It is shown that national interests combined with regional outbreaks significantly influence publication intensity. Thus, a correlation between national publication volume and the number of WNV cases was observed. In contrast to most life science topics, the scientific interest in WNV significantly decreased after 2006. The USA, as the main actor in WNV research, is at the centre of international networking. Recently, European countries are also getting involved according to their new-emerging outbreaks. The results demonstrate national interest in research activities with a lack of globally focused approaches that are urgently needed to better understand and assess the distribution and characteristics of WNV.

## Highlights

- There is a correlation between national WNV cases and the number of publications.
- The declining trend in WNV research does not correspond to the growing prevalence.
- USA as most-publishing country is also at the centre of networking WNV research.
- France is the primary network partner for African and Middle Eastern countries.

## Introduction

West-Nile virus (WNV) is a mosquito-borne arbovirus of the family Flaviviridae belonging to the Japanese encephalitis antigenic complex [1]. Until now, WNV spread around the world and caused human infections in every continent – except Antarctica [2]. Infections in humans are mainly associated with two lineages L1 and L2, which are particularly responsible for neuroinvasive diseases [3,4]. WNV also compromises the safety of blood transfusions and transplants and poses a threat to patients whose lives are at risk from acquired infection [5]. WNV lineage 1 is differentiated into two sub-lineages: 1A is mainly distributed in Africa, Europe, the Middle East, North America, and West-Asia and is associated with the highest pathogenicity for humans [6]; 1B (Kunjin) was isolated in Oceania and rarely causes neurological symptoms [7]. Lineage 2 is more prevalent in the Western hemisphere [8].

Mosquito members of the Culex (Cx.) genus, especially *Cx. pipiens*, *Cx. tarsalis*, *Cx. quinquefasciatus* are the main transmitting vectors [9]. A few cases have been transmitted by other causes such as blood transfusion [10], organ transplantation [11], breast milk [12], or even vertically [13]. In addition to mosquitoes, birds are natural hosts of WNV and maintain the virus in the transmission cycle. In the United States of America (USA), the American robin is the primary host [14,15]. Not all hosts are known yet [16]. As “dead-end” hosts, primarily humans and horses were evidenced, terminating the cycle of transmission [6]. The mosquito egg hatch is temperature-dependent, as is the external incubation period between the blood meal and the capacity to transmit the virus [17,18].

The biggest outbreaks of WNV infection have been reported in the USA, Greece, Russia, Romania, and Israel, mainly on major bird migration routes [19,20]. Infections also occurred in Algeria, Congo, Canada, Brazil, Mexico, and the Caribbean [21,22].

In the USA, seven million infections were estimated and more than 51,000 clinical cases with about 2300 human deaths were reported since 1999 [23].

In 20% of infections, the disease progression of WNV infections (West Nile Fever) in humans is relatively mild showing symptoms like muscle pain, headache, fatigue, nausea, rash, hyperthermia, and swollen glands. About 80% of infections show no symptoms at all but it can lead in one of 150 cases to severe diseases (West Nile Virus Neuroinvasive Disease) such as encephalitis and meningitis which can lead to paralyses or death [1,6]. Older or immunocompromised people have the highest risks of developing a severe disease [24].

There are no vaccines or virus-specific therapeutic options approved for humans. Only for horses, vaccines are available [1]. Until now, no human WNV vaccine has progressed beyond phase 1 or 2 in clinical trials [25]. Several factors have hindered the progression of these vaccines, including challenges in designing and implementing efficacy studies, concerns regarding vaccine safety, and the costs associated with WNV vaccine programs. Conducting randomized large-scale phase 3 clinical trials proves to be challenging due to the sporadic and unpredictable nature of WNV activity areas prior to the WNV season. Consequently, trial preparations such as obtaining ethical approval for vaccine efficacy trials before the onset of the WNV season are difficult to accomplish [25]. Therefore, vector-control is one of the measures to prevent or limit WNV outbreaks. Larviciding reduces the number of mosquito-vectors and serves as a method against the spread of infections. However, it has limited feasibility due to the inaccessibility of affected areas, resistance, and impact on non-target groups [26]. Adulticide interventions, e.g. aerial ultra-low volume spraying of insecticides, have been proven effective and should accompany other vector-control measures and public education campaigns aiming at the reduction of vector breeding sites, e.g. open water collections for gardening [26].

Up to now, the introduction and increase of WNV in endemic and non-endemic regions have been linked to climate change, travel, and globalization [27]. WNV infections are predicted to spread in currently temperate regions [28], as higher temperatures and precipitation in summer promote them [29].

Globally, this increases and magnifies the risk for people unaware of the potential dangers of WNV infection. In particular, travellers and people working outdoors are at risk and should be informed about the risks and measures for self-protection [30]. Global and improved surveillance programs are needed in many countries to reduce public health risks and better assess epidemiologic parameters [30]. The noted trend of declining research activity and funding is astonishing, given that WNV continues to cause mortality and morbidity now and in the future [23]. Calls to classify WNV as an officially recognized neglected tropical disease are growing because it meets World Health Organization (WHO) Category A criteria, as it affects vulnerable populations living in climates conducive to mosquito-borne diseases, and the infection is poorly addressed by clinicians, researchers, and policymakers [23].

Previous studies examined WNV publications using standard parameters such as the number of annual publications and country ranking up to 2016 [31,32]. The present study is extended to 2022 and, thus, includes years in which WNV cases occurred in colder regions due to newly immigrated vectors as a result of climate change. In addition, expanded parameters were applied to provide a deeper look at the WNV research landscape, taking into account socioeconomic and epidemiological aspects. This study is, therefore, the first in-depth analysis of WNV from these aspects of publication trends and dissemination, highlighting incentives, challenges, and imperatives for future research.

## Methods

The *Web of Science* (WoS) Core Collection serves as the default online database in all studies embedded in the *New Quality and Quantity Indices in Science* (NewQIS) methodological platform [33]. This platform combines standard bibliometric approaches with advanced methods for an in-depth analysis of the worldwide publication output of scientific topics. The methods applied include temporal and geographical aspects as well as socio-economic and epidemiological parameters of the global research output. These are combined with visualization techniques such as *Density Equalizing Map Projection* (DEMP) [34] and cluster visualization using VOSviewer 1.6.19 [35]. DEMPs are distorted maps that inflate or deflate country sizes according to the evaluation parameter until an equilibrium of density is reached in all countries. The VOSviewer creates clusters of nodes and connecting lines for all keywords used in the articles to show important foci of the studies performed.

To create a valid and representative database, the search term for the search for metadata of publications on WNV must be developed in such a way that as many relevant publications as possible are found and entries that do not fit the topic are excluded as far as possible. For this purpose, we used the following string as the title search: “west nil? virus*” OR “west nil? fever” OR “west nil? encephalit*” OR “wnv infection*” OR “Egypt 101” OR “Kunjin virus*.” The asterisk stands for any number of following characters, while the question mark stands for one or no following character distinguishing between the terms “West Nil” (German) or “West Nile” (English). The Boolean operator “OR” combines the different terms to search for all terms used. After downloading the entries, we filtered by the document type “article” to include only original papers on WNV in this study. The evaluation date was January 31, 2023, and no time limit was set. The metadata of the entries found were sorted according to the analysis parameters and recorded in an MS Access database to enable the subsequent analyses.

The retrieved metadata of the articles were analyzed for publication and citation counts per year, country, affiliation, and journals. In addition, the keywords of the articles and the WoS categories were analyzed. The WoS categories represent the assigned subject area of the journals listed in WoS, which is also assigned to each article published in the respective journal. In combination with the keywords, they were evaluated to show the relevant topics of WNV research by country and time. For inclusion of socio-economic aspects, the population size of the countries and their *Gross Domestic Product* (GDP) [36] were used and set in relation to the article numbers of the respective countries.

Data of human case numbers of WNV infections for epidemiological analyses were retrieved from the *European Centre for Disease Prevention and Control* (ECDC) for European Countries, Israel, Algeria, Tunisia, and Russia [37,38]. Additionally, the following sources for countries’ case numbers were used: *Centre of Disease Control and Prevention* (CDC) [39] for USA data, *Canadian Government Surveillance* for Canadian data [40], WHO *Regional Office for the Eastern Mediterranean* [41] for additional Tunisian data, the *Better Health Channel* of the Victorian Government [42], *Surveillance Report of Queensland* [43], *Annual Report of the National Arbovirus and Malaria Advisory Committee* [44] for Australia, epidemiological surveillance data for Brazil [45,46], and the *Health Protection Surveillance Centre, Ireland* for Irish data [47].

Equine epidemiology data were not included in the global country analyses because comparable country-level data are limited, so validated analyses could not be performed.

## Results

Metadata of 3933 original articles (*n*) on WNV could be in the analysis database by applying the developed search strategy.

### Chronological aspects

**Figure 1.**
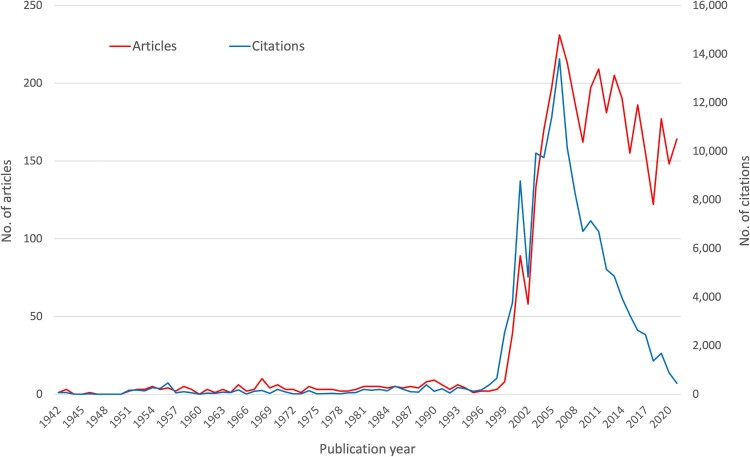
Annual article numbers and annual citation numbers over time (1942–2022).

The first publication found was an article published in Uganda in 1942, which dealt with the differentiation of WNV from St. Louis virus and Japanese encephalitis virus [48]. The next decades were characterized by single-digit numbers of articles per year. It was not until 1968 that ten articles were published on WNV. Annual publication numbers increased significantly in 2000 and reached a maximum in 2006 with *n* = 13,729 articles. The development of the annual citation numbers was similar, with the highest value also in 2006 and a subsequent decrease (Figure 1).

Analogous to the annual numbers, the publication years of the most frequently cited articles on WNV were between 1999 and 2006. Only two articles in the top ten were published outside this period. One was from 1998 and dealt with the Romanian WNV outbreak in 1996 and another from 2009 dealt with the role of interferon-inducible transmembrane (IFITM) proteins in the cellular resistance of some viruses. The other highly-cited articles were mostly focused outbreaks of WNV in the USA and Europe (Supp. Table 1).

### Geographical aspects

Of all 3933 articles, *n* = 3833 articles could be assigned to a country of origin (97.43%) and thus used to analyze countries’ publication efforts on WNV.

**Figure 2.**
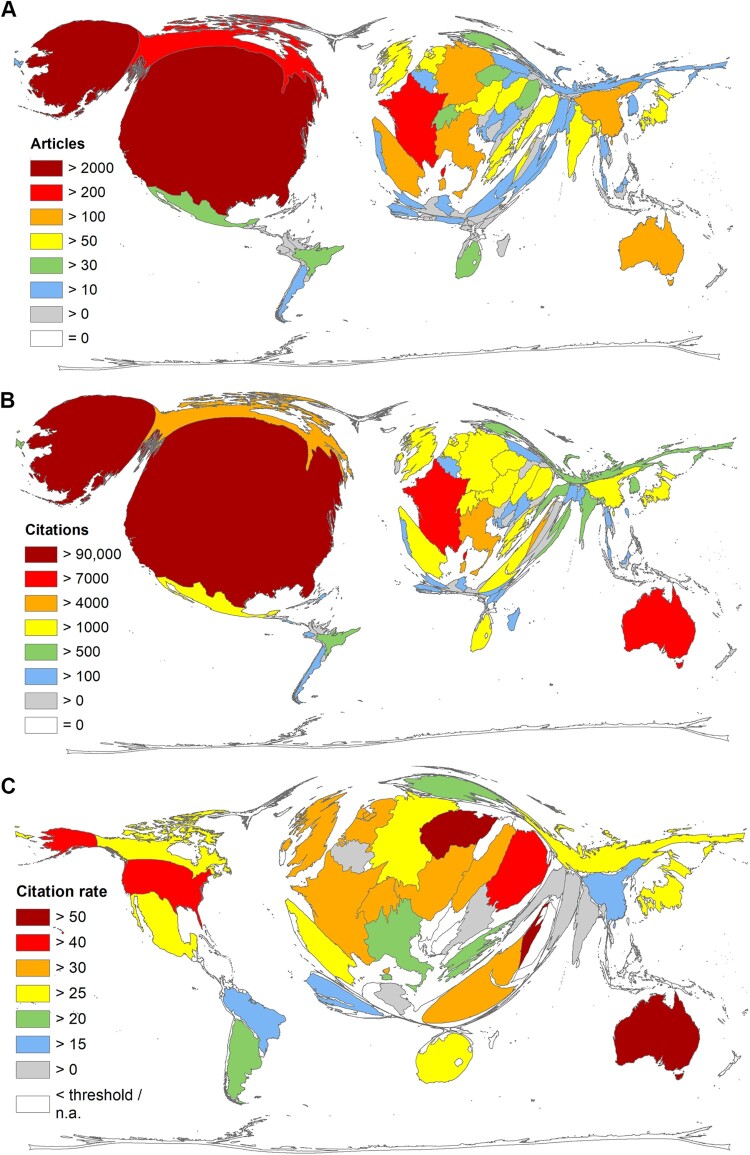
Density equalizing map projections. (A) Number of articles. (B) Number of citations. (C) Citation rate (number of citations/number of articles). Analysis threshold ≥ 20 articles on WNV.

The USA published by far the most publications on WNV with *n* = 2205 articles, followed by Canada (*n* = 225), France (*n* = 209), Italy (*n* = 192), and Germany (*n* = 151). China ranks 10th (*n* = 106), and the United Kingdom (UK) ranks 11th (*n* = 98) (Figure 2(A)). In terms of the number of citations (c) countries received for WNV publications, another ranking of the top five countries emerges: USA (*c* = 97,711), Australia (*c* = 10,526, *n* = 187), France (*c* = 7959), Canada (*c* = 5666), and Israel (*c* = 4625, *n* = 88) (Figure 2(B)). Chinese articles reached *c* = 1944, ranking the country 17th. Considering the citation rate (cr = number of citations / number of articles) of countries with at least 20 publications on WNV (analysis threshold), the ranking is as follows: Australia (cr = 56.29), Czech Republic (cr = 55.57), Israel, (cr = 52.56), Romania (cr = 48.55), and Singapore (cr = 46.02) (Figure 2(C)).

Considering the development of countries’ publication on WNV research over time, the analysis of the relative share of the most publishing countries reveals the increased of publication efforts by countries other than the major player USA – but at a relative low level (Suppl. Figure 1).

### Socio-economic aspects

**Figure 3.**
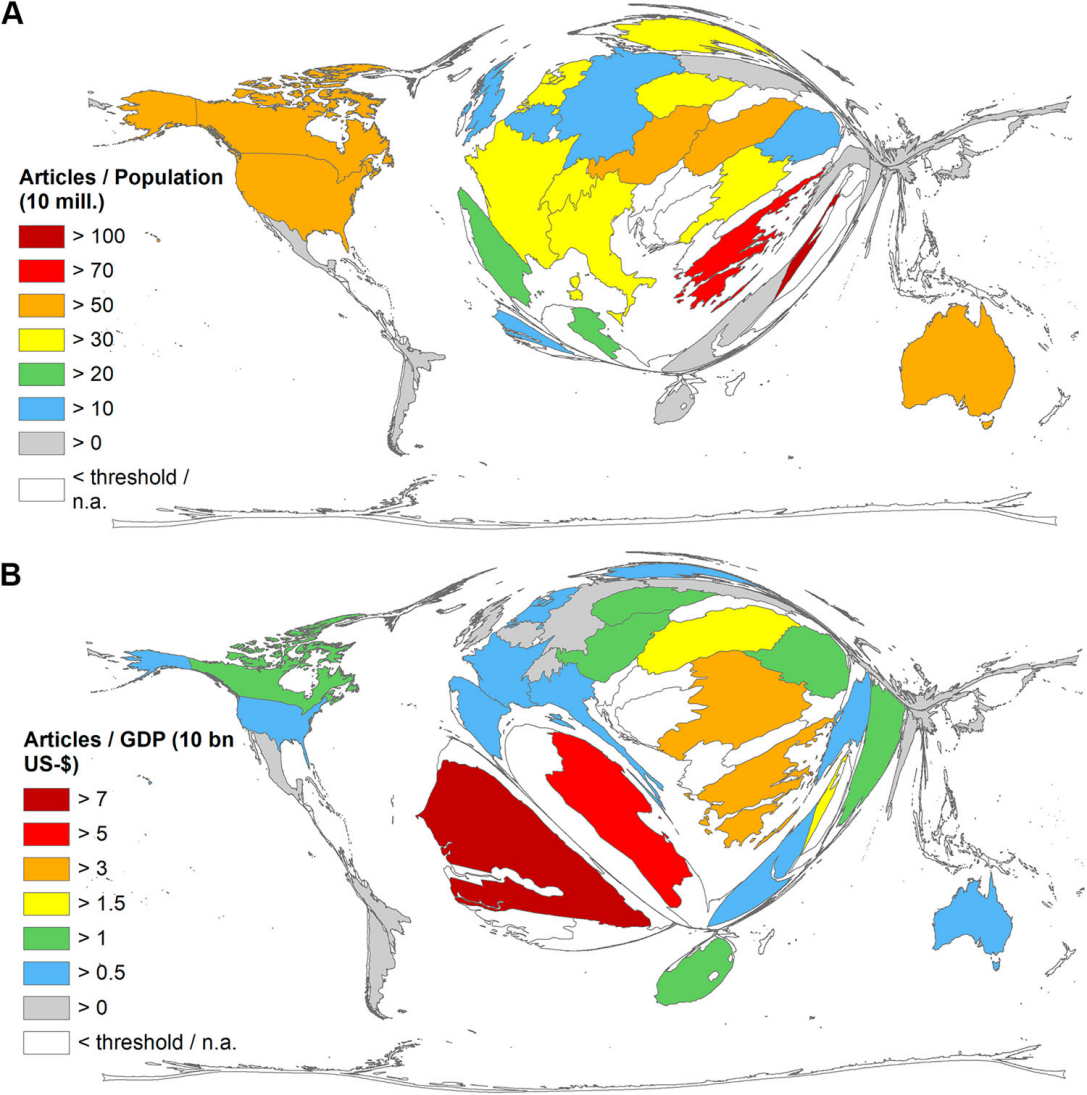
Density equalizing map projection (DEMP) of socio-economic parameters. Analytical threshold ≥ 20 articles on West-Nile Virus. (A) R_POP_: Number of articles/population in 10 mill. inhabitants, R_GDP_: Number of articles/GDP (Gross Domestic Product) in $10 billion.

When analyzing the relationship between the number of articles and the socioeconomic characteristics of the countries with at least 20 articles on WNV (analytical threshold), a different cartogram of the world map emerges. In terms of R_POP_ (number of articles/population size [10 million inhabitants]), the leading country was Israel (R_POP_ = 100.11), followed by Singapore (R_POP_ = 99.03), Greece (R_POP_ = 84.85), Australia (R_POP_ = 72.51), and the USA (R_POP_ = 66.44) (Figure 3(A)). Looking at the ratio of articles to gross domestic product (GDP) in $10 billion (R_GDP_), the rankings are as follows: Senegal (R_GDP_ = 7.96), Tunisia (R_GDP_ = 5.55), Serbia (R_GDP_ = 4.76), Greece (R_GDP_ = 4.07), and Hungary (R_GDP_ = 2.80) (Figure 3(B)).

The supplementary Table 2 shows all R_GDP_ and R_POP_ values and the ranking of countries ≥ 20 articles on WNV.

### Epidemiological aspects

For the calculation of the ratio of epidemiological parameters – here: Number of human cases per country – only a few country data were accessible, mainly from European and North American countries covered by regional surveillance programs.

In the USA, the number of confirmed human WNV cases (24,741 cases) from 2011 to 2021 was more than ten times higher than in the country with the second-highest recorded number of cases, Canada (1904 cases). Highly affected countries also include Russia (1786 cases), Greece (1506 cases), Serbia (1472 cases), and Italy (1246 cases), with more than 1000 cases of WNV infection in humans. When the number of articles is related to the number of human cases (R_Cases_), Australia ranks first (R_Cases_ = 49.67), followed by Egypt (R_Cases_ = 29), Portugal (R_Cases_ = 12), Slovakia (R_Cases_ = 129), and the Netherlands (R_Cases_ = 9.14) (Table 1).

**Table 1.** Country ranking of the ratio of the number of articles and the number of human cases of human WNV infections per country (R_Cases_).

Country	Articles	Cases^a^	R_Cases_
Australia	149	3	49,67
Egypt	29	1	29,00
Portugal	12	1	12,00
Slovakia	12	1	12,00
Netherlands	64	7	9,14
Germany	151	22	6,86
France	205	31	6,61
Czech Republic	42	8	5,25
Ireland	5	1	5,00
Algeria	5	2	2,50
Spain	172	86	2,00
Austria	60	46	1,30
Turkey	62	48	1,29
Albania	4	4	1,00
Slovenia	3	5	0,60
Palestine	3	7	0,43
North Macedonia	12	32	0,38
Cyprus	5	18	0,28
Montenegro	2	10	0,20
Croatia	16	101	0,16
Italy	192	1246	0,15
Canada	224	1904	0,12
Hungary	51	468	0,11
Israel	88	808	0,11
Bulgaria	3	30	0,10
Ukraine	4	43	0,09
Kosovo	2	22	0,09
USA	2202	24741	0,09
Romania	31	457	0,07
Greece	88	1506	0,06
Tunisia	26	522	0,05
Bosnia & Herzegovina	1	32	0,03
Serbia	28	1472	0,02
Russia	26	1786	0,01
Syria	0	2	0,00

^a^ Sum of confirmed cases from 2011 to 2021 [37–47].

**Figure 4.**
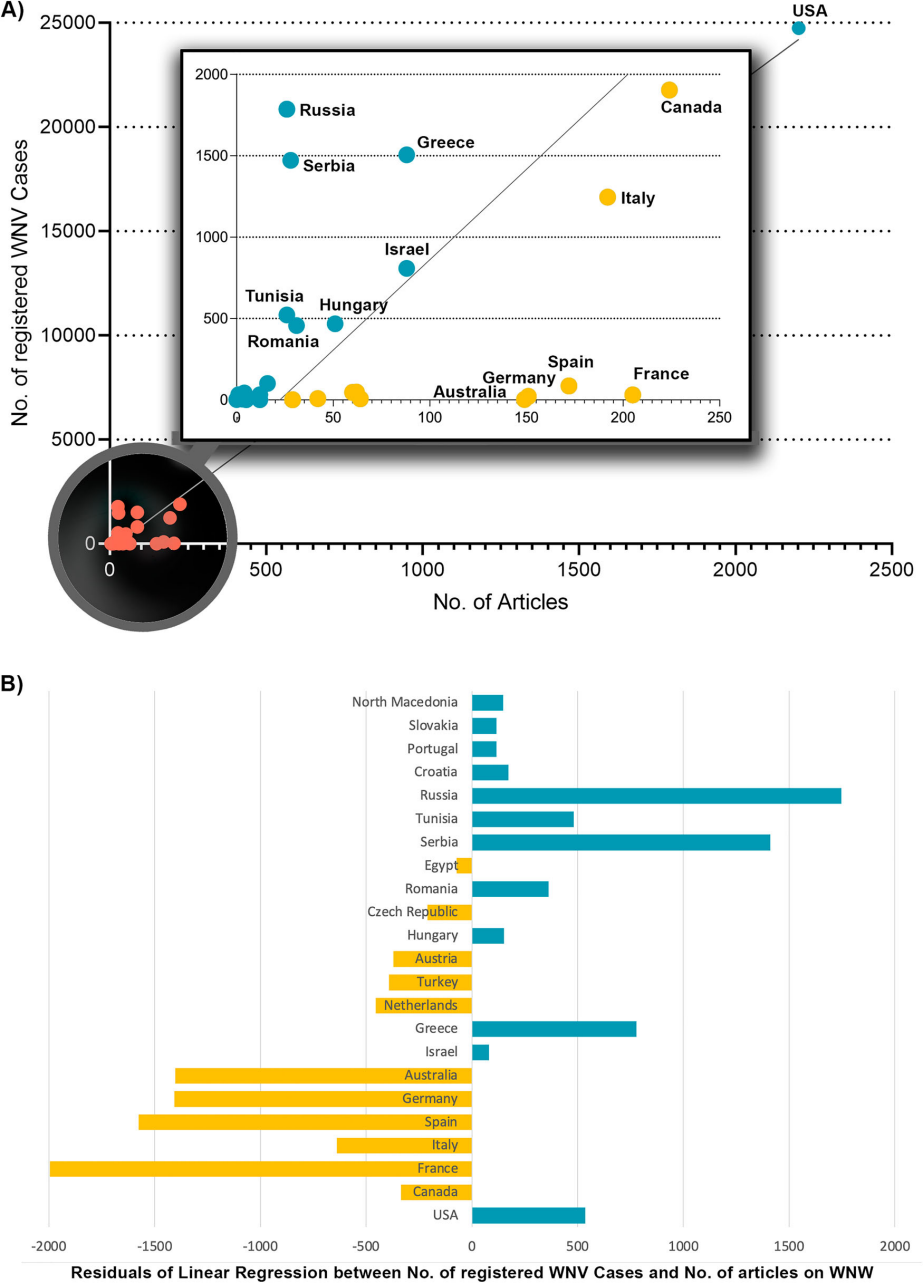
Linear regression between the number of registered human West-Nile Virus (WNV) cases [37–47] and the number of articles on WNV per country. Inlet: additional magnifying glass display for countries excluding the USA. (A) Spearman regression (*r*^2^ = 0.97). (B) Residuals of countries with more than 10 articles on WNV (threshold): Negative residuals (yellow) are below the regression line, i.e. there are relatively more articles about WNV in relation to registered human WNV cases. Positive residual values (blue) are above the regression line, i.e. there are relatively fewer articles about WNV in relation to the registered human WNV cases.

Linear regression (*r*^2^ = 0.97) and Spearman correlation (*p* = 0.0021 **) of the number of registered cases and the number of articles per country with nonparametric distribution were significant (Figure 4(A)). The display of the residuals of the linear regression distinguished between countries that were relatively more engaged in WNV articles and those that were comparatively behind in terms of registered cases (Figure 4(B)).

Table 2 lists the countries with the first occurrences of autochthonous human WNV cases.

**Table 2.** Time table of the first autochthonous human West-Nile Virus (WNV) cases.

Country	Year of first autochthonous human WNV case	Reference
Greece	2010	Papa et al. (2010) ^2^
UK	None	NHS (2020) ^3^
China	2004	Li et al. (2013) ^4^
Spain	2004	Kaptoul et al. (2007) ^5^
Australia	1992	Phillips et al. (1992) ^6^
Germany	2018	Ziegler et al. (2019) ^7^
Italy	2011	Bagnarelli et al. (2011) ^8^
France	2003	Mailles et al. (2003) ^9^
Canada	2002	Pepperell et al. (2003) ^10^
USA	1999	Lanciotti et al. (1999) ^11^

### Research fields

**Figure 5.**
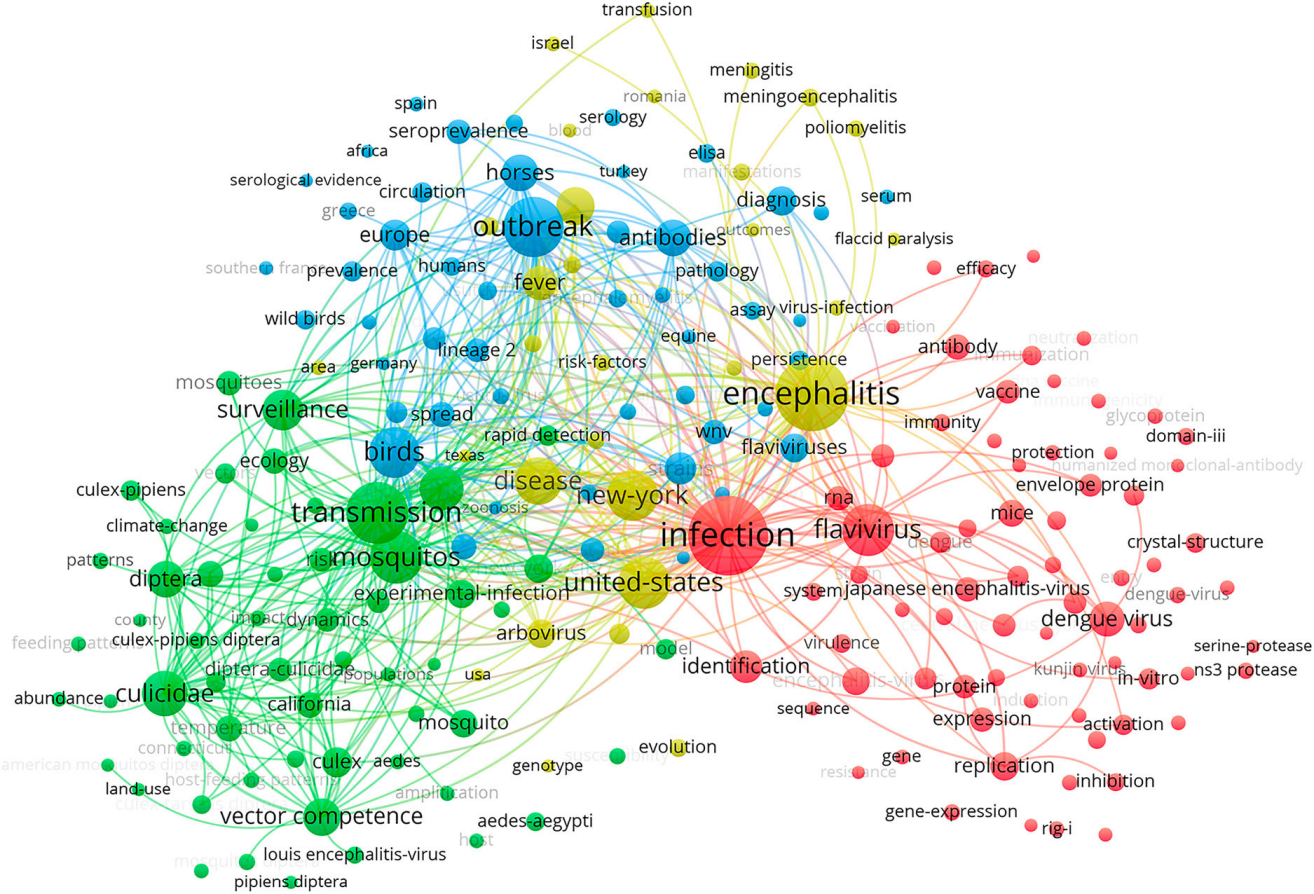
Clusters of keywords. Red cluster: identification, green cluster: transmission, blue cluster: epidemiological topics, yellow cluster: health topics.

Cluster analysis of the keywords resulted in four thematic groups: Identification, transmission, epidemiology, and health-related topics (Figure 5).

**Figure 6.**
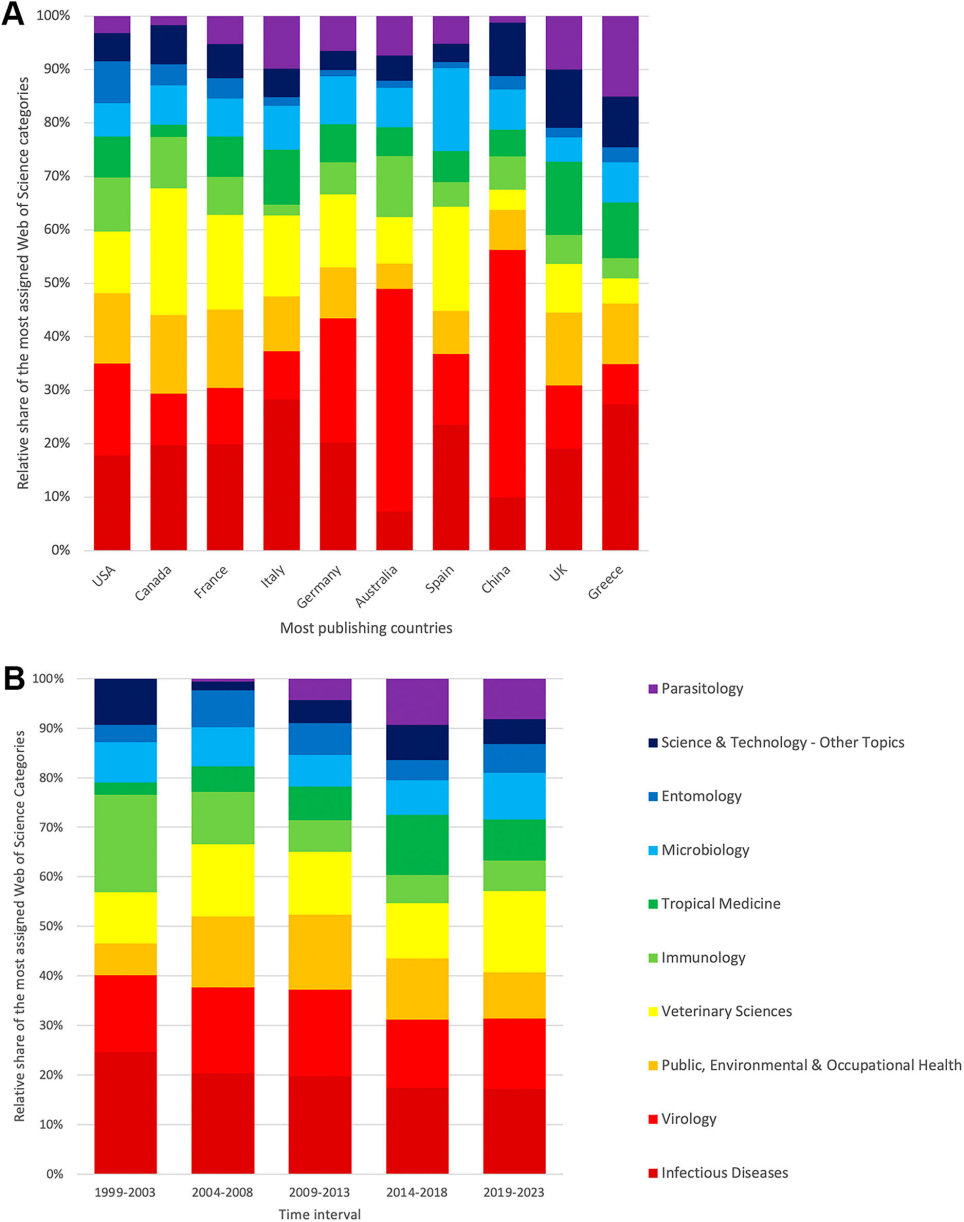
Most assigned Web of Science research areas. (A) Geographical distribution: Relative share in the ten most publishing countries. (B) Evolution over time – Relative share in 5-year intervals from 1999 to 2023.

Analysis of the assigned Web of Science (WoS) research areas revealed the following order of the top five: *Infectious Diseases* (*n* = 808), *Virology* (*n* = 707), *Public, Environmental & Occupational Health* (*n* = 554), *Veterinary Sciences* (*n* = 551), and *Immunology* (*n* = 368). The distribution varies in some countries. In contrast to the relatively even distribution of categories in most of the top publishing countries, the research area *Virology* dominates at the expense of *Infectious Diseases* in China and Australia (Figure 6(A)).

The relative proportion of the most frequently assigned WoS area also varied over time (Figure 6(B)), showing the decline of *Immunology* (19.70%: first interval to 6.27%: last interval) and onset and increase of *Parasitology* (0%: first interval to 8.20%: last interval). *Veterinary Sciences* also increased over time, reaching second place in the last interval from 2019 to 2023 (16.40%), just behind *Infectious Diseases* (17.20%).

### International networking

Collaboration between countries is defined by the country of origin of the authors’ affiliation, with at least two countries involved in an article. At the international level, an extensive network concerning WNV research has developed, with the USA as the core country. The USA collaborated primarily with Canada (*n* = 55), followed by Australia (*n* = 38), China (*n* = 35), France (*n* = 31), Mexico (*n* = 30), and the UK (*n* = 27). The most frequently published bilateral partnerships without US participation were Germany/Italy (*n* = 19), Austria/Hungary (*n* = 17), and Greece/Italy (*n* = 17) (Suppl. Figure 2).

### Publishing institutions

At the level of publishing institutions, most publications on WNV came from the *US Centres for Disease Control and Prevention* (CDC) (*n* = 320, *c* = 20,472), followed by the *New York State Department of Health* (*n* = 154, *c* = 11,680), various US universities, and the *National Institutes of Health* (NIH). The most publishing non-US institutions include the *University of Thessaloniki* (Greece), *Institute Pasteur* (France), and INIA (*Instituto Nacional de Investigación y Tecnología Agraria y Alimentaria*, Spain). Yale University (USA) achieved the highest citation rate (cr = 91.73), followed by Washington University (cr = 88.82) (Supp. Figure 3).

The collaborations were mainly between institutions in one country. The most fruitful collaboration was between the *New York State Department of Health* and SUNY (*State University New York*) at Albany, resulting in 66 articles on WNV. The US CDC collaborated mainly with *Colorado State University* (*n* = 48). The partnership between the Greek CDC and *Aristotle University* in Thessaloniki, Greece, was that with the most publications from non-US institutions. The most extensive binational collaboration was between the *University of Veterinary Medicine Vienna* (Austria) and the *Academy of Sciences* (Czech Republic), with *n* = 9 joint articles on WNV.

## Discussion

The generated database with bibliometric information on 3933 original articles on WNV spans the time period from 1943 to January 2023. Although WNV was first isolated in Uganda as early as 1937 [23], the first article listed in WoS dates from 1942 [48]. It is a study on the differentiation of WNV written by K.C. Smithburn, who served as director of the Yellow Fever Research Institute in Uganda. His career was based primarily on virus isolation, especially the Yellow Fever virus [49]. In an article published as early as 1940, his team described the neurotropic virus and named it West Nile virus after the region from which the first patient came [50]. This article was published in the *American Journal of Tropical Medicine & Hygiene*, whose articles have been listed at WoS only since 1952. Therefore, this analysis could not capture this initial virus description and its symptoms. Worldwide scientific interest was subsequently very low reaching a significant increase in the number of annual articles and their citations only with the registration of about 400 infections in Romania in 1998 and with the outbreak in the USA in 1999. This increased interest was highlighted by the publication of the most cited articles in this study by Tsai et al. [51] and by Lanciotti et al. [52]. Shortly after, new cases and outbreaks were also reported in other European countries [53], which also contributed to the rapidly growing scientific interest during this period. After a first maximum of article numbers and their citations in 2001, the highest values were reached in 2006. The extreme decrease of citations afterward is partly due to the short time that the respective articles have had to generate more citations, and partly due to the waning interest in research activities, as evidenced by the fluctuating downward trend in annual article numbers since then. This trend is characterized by some spikes indicating outbreaks mainly in Eastern European countries but also in Western Europe (Italy and Spain), where a higher number of cases was also registered [37]. We could show a significant correlation between the number of registered cases and the research activity on a national level (Figure 4). In addition to the generally high research performance of the USA, this of course pushes them far ahead into first place regarding absolute publication numbers. That also applies to the European countries concerned. The largest outbreaks have been reported in the USA, Russia, Romania, Israel, and Greece, primarily located along bird migration routes [6], conditioning their interest in WNV research. Following our findings, the USA was also shown as the major player in a bibliometric study analyzing publications on WNV until the year 2016 in the Scopus literature database, followed by Canada, France, Italy, and Germany [31]. This analysis shows a sharp increase in annual publications starting in 2000, peaking in 2006. Another study, also using Scopus and evaluating the same time period, shows the same temporal development but a different ranking of the five leading publishing countries: the USA, France, Canada, Italy, and Australia [32]. When analyzing the citation rates, a different picture emerges: Australia and Israel position themselves as the leading countries. They were involved, in collaboration with the USA and France, in the most cited article from 1998, which reached 1165 citations by the time of the evaluation and provided the genome sequencing that pointed to the responsibility of WNV for the human encephalitis outbreak in the USA [52]. This dramatic outbreak was imported from viruses circulating in Israel and Tunisia [6]. In 2003, an annual maximum of 9862 cases were registered in the USA [39]. Interest also stimulated Australian research on the Kunjin subtype [54] and the outbreak in Israel in 2000, when 417 human cases and 326 hospitalizations were reported [55]. Israel led also in terms of the demographic ratio (R_POP_), followed by Singapore in second place that could also be highlighted regarding its citation rate. Singaporean researcher M.L. Ng collaborated with Australian virologist E.G. Westaway on the Kunjin virus, which sparked national interest in WNV research in the 1980s [56]. In addition to this link, the *Novartis Institute for Tropical Diseases* in Singapore and the *Novartis Institute for Biomedical Research* in Switzerland also contributed to the Singaporean and Swiss ranking in WNV research [57].

Looking at the countries with at least 20 articles on WNV (threshold value), Senegal and Tunisia are at the top of the economic indicators. Both countries are only just above the threshold value. Together with their low GDPs, the top position is explainable. Nevertheless, their performance regarding other research areas is comparably high [33], pointing to the outbreak in Tunisia with 377 reported human cases in 2018 [41]. Senegalese WNV research is conducted above all by the *Institute Pasteur* in Dakar, which collaborates with the *Institute Pasteur* in France, which in turn is among the most publishing institutions in the field of WNV, consisting mainly of US institutions. The USA is the core of the international WNV research network, dominated by Canadian, Australian, Chinese, and French partnerships (Figure 5). The countries of the Middle East and North Africa cooperate with the USA only to a very limited extent. Instead, France is the most important partner country here. This points to the historical relationship of these countries, e.g. their former French colonial status, and the associated establishment of foreign institutes to address medical problems in developing countries with a synergy effect of all member countries [58]. Harnessing synergies for a broader scientific network between high-income countries and low- and middle-income countries would lead to more sophisticated approaches that are global in scope rather than national in focus.

The identified risk of co-circulation of WNV strains combined with the diversity of WNV-competent vectors leads to an urgent call for more targeted research. It is not only in Africa or other developing regions that epidemiological characteristics, genetic variability, and distribution patterns are not fully known or quantified [59,60]. Even in European countries where co-circulation has recently been identified, routine diagnostic and screening methods or regulations are outdated [5].

The results of this study indicate that more global, targeted approaches and the establishment of a broader, more balanced network are needed for the development of sufficient strategies to address the increasing spread and associated risks of WNV infection.

## Conclusions

In summary, this study provides a bibliometric overview on global WNV research trends updated to the year 2022. The pioneering role of US-American researchers, triggered by the 1999 WNV outbreak in North America, started an increase in research outputs. The number of publications since the year 2000 is fluctuating, mainly depending on regional WNV outbreaks. While the publication output on WNV of the USA is decreasing, other countries such as Australia, China and European countries increased their percentage count on WNV publications due to their direct involvement shown through the registered human WNV cases. In addition to publication topics such as molecular pathways of WNV infection, outbreak and case reports, ecological features of virus-vector-host interaction, and prevention and vector control measures are mentioned in the keywords.

As advanced work, we suggest to substantively look closer into the development of these WNV research topics over time. WNV research topics associated with (A) project funding, (B) legal frameworks of WNV documentation, surveillance and monitoring or (C) evaluation of applied control methods can provide information about the status of preparedness of countries, which would be valuable information for political decision making and future research.

## Geological information

This study includes the global data of all countries that have published on mpox up to the time of the evaluation.

## Supplementary Material

Supplemental Material

## Data Availability

The data underlying this article will be shared with the corresponding author upon reasonable request, provided the recipient has a Web of Science license.
