# Supplementary material for: A virus becomes a global concern: research activities on West-Nile virus
Source: Emerg Microbes Infect. 2023 Sep 13;12(2):2256424. doi: 10.1080/22221751.2023.2256424 (PMC10501173; doi:10.1080/22221751.2023.2256424)

Supplement

**A Virus becomes a Global Concern: research activities on West-Nile Virus**

by

Doris Klingelhöfer, Markus Braun, Isabelle M. Kramer, Friederike Reuss, Ruth Müller, David A. Groneberg^1^, and Dörthe Brüggmann

**Supplementary tables**

Supplementary table 1: Most-cited articles on West-Nile Virus.

| **Author (country)** | **Year** | **Citations** | **Title** | **Journal** |
| --- | --- | --- | --- | --- |
| R.S. Lanciotti et al. [1]  (USA, France, Australia, Israel) | 1999 | 1165 | Origin of the West Nile virus responsible for an outbreak of encephalitis in the northeastern United States | Science |
| A.L. Brass et al. [2] (USA, UK) | 2009 | 923 | The IFITM Proteins Mediate Cellular Resistance to Influenza  A H1N1 Virus, West Nile Virus, and Dengue Virus | Cell |
| Nash, D et al. [3] (USA) | 2001 | 866 | The outbreak of West Nile virus infection  in the New York City area in 1999. | NEJM |
| N. Komar et al. [4] (USA) | 2003 | 852 | Experimental infection of north American birds  with the New York 1999 strain of West Nile virus | Emerg Infect Dis |
| T. Wang et al. [5] (USA) | 2004 | 844 | Toll-like receptor 3 mediates West Nile virus entry  into the brain causing lethal encephalitis | Nat Med |
| R.S. Lanciotti et al. [6]  (USA) | 2000 | 841 | Rapid detection of West Nile virus from human clinical  specimens, field-collected mosquitoes, and avian samples  by a TaqMan reverse transcriptase-PCR assay | J Clin Microbiol |
| Z. Hubalek, J. Halouzka [7] (Czech Republic) | 1999 | 769 | West Nile fever -  a reemerging mosquito-borne viral disease in Europe | Emerg Infect Dis |
| E.B. Hayes et al. [8] (USA) | 2005 | 706 | Epidemiology and transmission dynamics  of West Nile Virus disease | Emerg Infect Dis |
| Tsai et al. [9] (USA, Romania, France) | 1998 | 545 | West Nile encephalitis epidemic  in southeastern Romania | Lancet |
| M.J. Turell et al. [10] (USA) | 2005 | 470 | An update on the potential of North American mosquitoes  (Diptera: Culicidae) to transmit West Nile virus | J Med Entomol |

**References**

1. Lanciotti RS, Roehrig JT, Deubel V, et al. Origin of the West Nile virus responsible for an outbreak of encephalitis in the northeastern United States. Science. 1999 Dec 17;286(5448):2333-2337.

2. Brass AL, Huang IC, Benita Y, et al. The IFITM Proteins Mediate Cellular Resistance to Influenza A H1N1 Virus, West Nile Virus, and Dengue Virus. Cell. 2009 Dec 24;139(7):1243-1254.

3. Nash D, Mostashari F, Fine A, et al. The outbreak of West Nile virus infection in the New York City area in 1999. New Engl J Med. 2001 Jun 14;344(24):1807-1814.

4. Komar N, Langevin S, Hinten S, et al. Experimental infection of north American birds with the New York 1999 strain of West Nile virus. Emerg Infect Dis. 2003 Mar;9(3):311-322.

5. Wang T, Town T, Alexopoulou L, et al. Toll-like receptor 3 mediates West Nile virus entry into the brain causing lethal encephalitis. Nat Med. 2004 Dec;10(12):1366-1373.

6. Lanciotti RS, Kerst AJ, Nasci RS, et al. Rapid detection of West Nile virus from human clinical specimens, field-collected mosquitoes, and avian samples by a TaqMan reverse transcriptase-PCR assay. J Clin Microbiol. 2000 Nov;38(11):4066-4071.

7. Hubalek Z, Halouzka J. West Nile fever - a reemerging mosquito-borne viral disease in Europe. Emerg Infect Dis. 1999 Sep-Oct;5(5):643-650.

8. Hayes EB, Komar N, Nasci RS, et al. Epidemiology and transmission dynamics of West Nile Virus disease. Emerg Infect Dis. 2005 Aug;11(8):1167-1173.

9. Tsai TF, Popovici F, Cernescu C, et al. West Nile encephalitis epidemic in southeastern Romania. Lancet. 1998 Sep 5;352(9130):767-771.

10. Turell MJ, Dohm DJ, Sardelis MR, et al. An update on the potential of North American mosquitoes (Diptera : Culicidae) to transmit West Nile virus. J Med Entomol. 2005 Jan;42(1):57-62.

Supplementary table 2: Socio-economic parameters [11] of countries with at least 20 articles on WNV (threshold), sorted by R_GDP_. R_GDP_ = Number of articles / GDP (Gross Domestic Product in 10 bn US-Dollars), R_POP_ = Number of articles / Population in mill. inhabitants.

| **Country** | **Articles** | **GDP** in 10 bn US-Dollars | **R_GDP_** | **Rank  R_GDP_** | **Population** in 10 mill. | **R_POP_** | **Rank  R_POP_** |
| --- | --- | --- | --- | --- | --- | --- | --- |
| Senegal | 22 | 2.76 | 7.96 | 1 | 1.72 | 12.79 | 22 |
| Tunisia | 26 | 4.68 | 5.55 | 2 | 1.19 | 21.78 | 17 |
| Serbia | 26 | 6.31 | 4.44 | 3 | 0.68 | 40.91 | 9 |
| Greece | 88 | 21.62 | 4.07 | 4 | 1.04 | 84.85 | 3 |
| Hungary | 51 | 18.23 | 2.80 | 5 | 0.96 | 52.94 | 8 |
| Israel | 88 | 48.16 | 1.83 | 6 | 0.88 | 100.11 | 1 |
| Czech Republic | 42 | 28.23 | 1.49 | 7 | 1.07 | 39.16 | 10 |
| Singapore | 54 | 39.70 | 1.36 | 8 | 0.55 | 99.03 | 2 |
| Austria | 60 | 47.71 | 1.26 | 9 | 0.90 | 66.35 | 6 |
| Australia | 187 | 154.27 | 1.21 | 10 | 2.58 | 72.51 | 4 |
| Iran | 28 | 23.15 | 1.21 | 11 | 8.50 | 3.29 | 27 |
| Canada | 225 | 199.08 | 1.13 | 12 | 3.81 | 59.10 | 7 |
| Romania | 31 | 28.41 | 1.09 | 13 | 1.91 | 16.21 | 20 |
| South Africa | 44 | 41.99 | 1.05 | 14 | 6.00 | 7.33 | 23 |
| USA | 2205 | 2299.61 | 0.96 | 15 | 33.19 | 66.44 | 5 |
| Italy | 192 | 209.99 | 0.91 | 16 | 6.04 | 31.81 | 13 |
| Spain | 127 | 142.53 | 0.89 | 17 | 4.67 | 27.17 | 16 |
| Turkey | 62 | 81.53 | 0.76 | 18 | 8.50 | 7.29 | 24 |
| Egypt | 29 | 40.41 | 0.72 | 19 | 10.43 | 2.78 | 29 |
| France | 209 | 293.75 | 0.71 | 20 | 6.75 | 30.97 | 15 |
| Netherlands | 64 | 101.80 | 0.63 | 21 | 1.72 | 37.27 | 11 |
| Sweden | 32 | 62.74 | 0.51 | 22 | 1.02 | 31.50 | 14 |
| Argentina | 23 | 49.15 | 0.47 | 23 | 4.56 | 5.04 | 26 |
| Switzerland | 32 | 81.29 | 0.39 | 24 | 0.87 | 36.72 | 12 |
| Belgium | 23 | 59.99 | 0.38 | 25 | 1.16 | 19.77 | 18 |
| Germany | 151 | 422.31 | 0.36 | 26 | 8.39 | 18.00 | 19 |
| Mexico | 42 | 129.30 | 0.32 | 27 | 13.03 | 3.22 | 28 |
| Brazil | 35 | 160.90 | 0.31 | 28 | 21.40 | 1.64 | 31 |
| UK | 98 | 318.69 | 0.30 | 29 | 6.82 | 14.37 | 21 |
| India | 65 | 317.34 | 0.20 | 30 | 139.34 | 0.47 | 33 |
| Russia | 26 | 177.58 | 0.15 | 31 | 14.59 | 1.78 | 30 |
| Japan | 66 | 493.74 | 0.13 | 32 | 12.57 | 5.25 | 25 |
| China | 106 | 1773.41 | 0.06 | 33 | 144.42 | 0.73 | 32 |

11. UIS.Stat. Data 2017, URL: <http://data.uis.unesco.org/Index.aspx> (accessed Nov 2019). 2022.

**Supplementary figures**

Supplementary figure 1: Development of the relative share of the ten most publishing countries in 4-year intervals from 1999 until 2022.


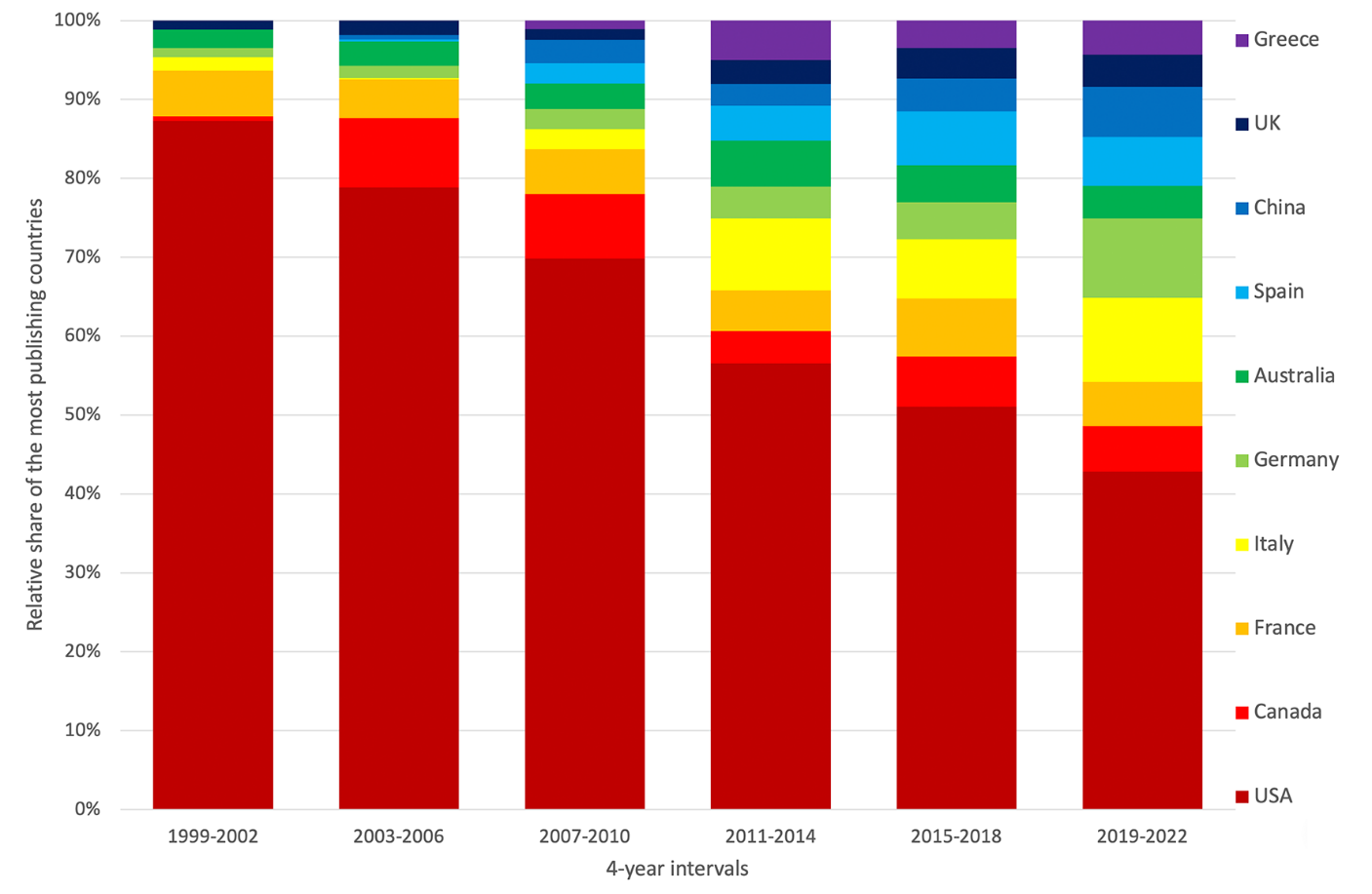


Supplementary figure 2: International collaboration network. Display threshold: > 3 collaboration articles between two countries on West-Nile Virus (WNV). Countries that did not have more than two partnerships with the USA were placed outside the circle. Numbers in brackets (number of articles on WNV / number of collaboration articles on WNV).


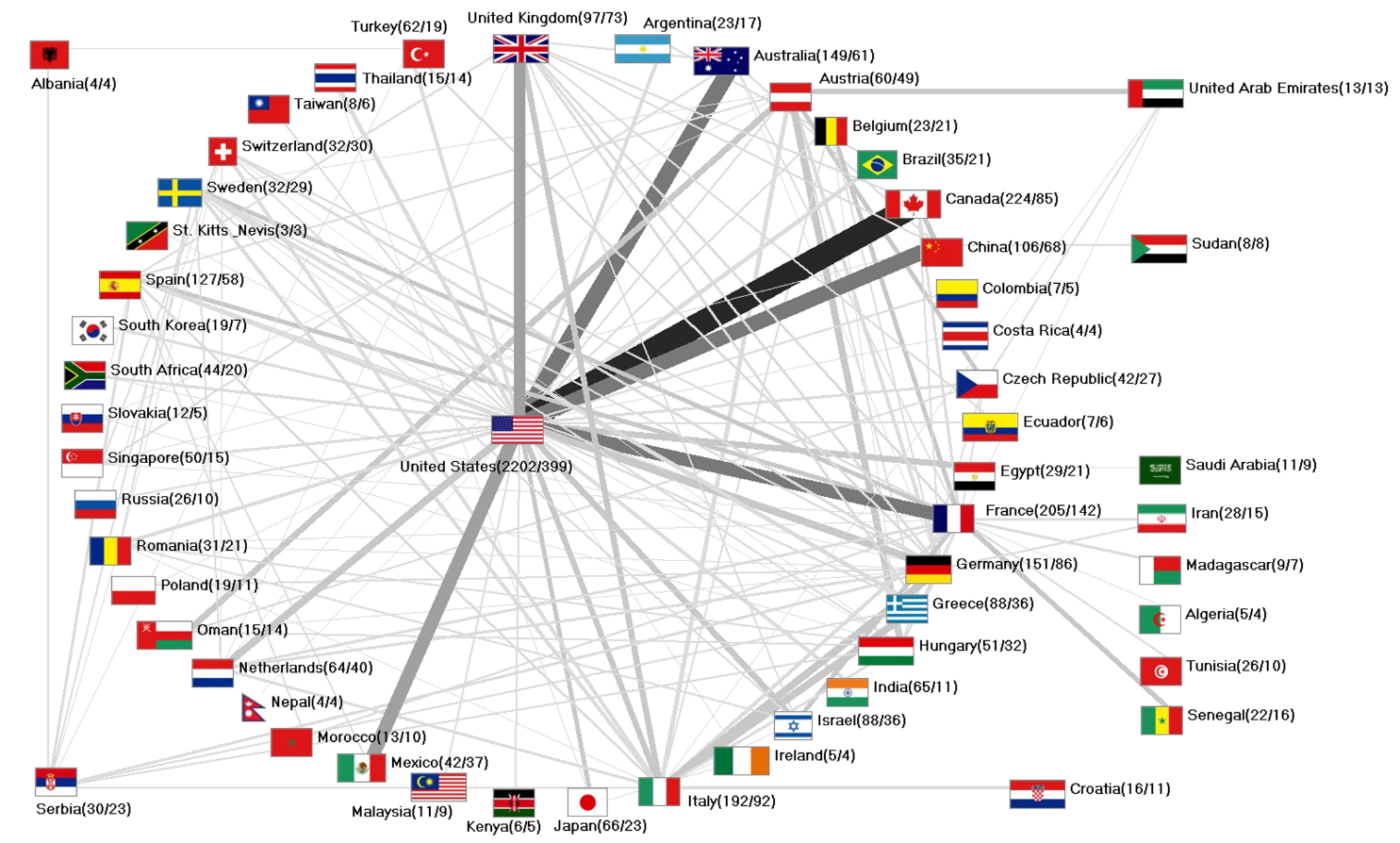


Supplementary figure 3: Most publishing institutions, INIA: Instituto Nacional de Investigación y Tecnología Agraria y Alimentaria, CAES: The Connecticut Agricultural Experiment Station, USDA: U.S. Department of Agriculture, NIH: National Institutes of Health, SUNY: State University New York, CDC: Center of Disease Control and Prevention.


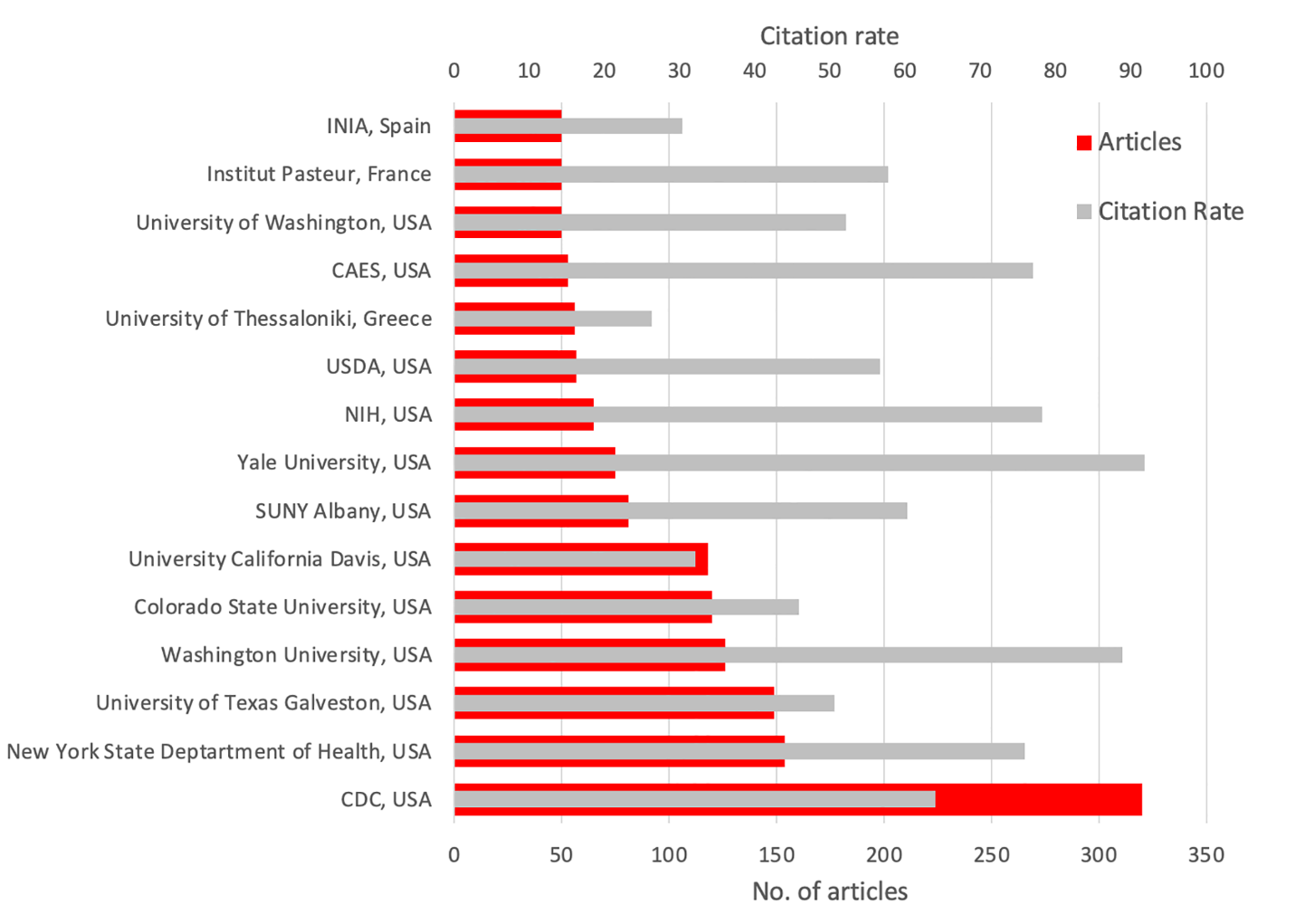

Supplement: Supplemental Material [file TEMI_A_2256424_SM8795.docx]
